# Correlation of *MLH1* and *MGMT* expression and promoter methylation with genomic instability in patients with thyroid carcinoma

**DOI:** 10.1186/1471-2407-13-79

**Published:** 2013-02-15

**Authors:** Juliana Carvalho Santos, André Uchimura Bastos, Janete Maria Cerutti, Marcelo Lima Ribeiro

**Affiliations:** 1 Unidade Integrada de Farmacologia e Gastroenterologia, Universidade São Francisco, Av. São Francisco de Assis, 218. Jd. São José, Bragança Paulista, SP, Brazil; 2 Laboratório as Bases Genéticas dos Tumores da Tiroide, Disciplina de Genética, Universidade Federal de São Paulo, São Paulo, SP, Brazil

**Keywords:** Microsatellite instability, DNA methylation, DNA repair, Thyroid carcinoma

## Abstract

**Background:**

Gene silencing of the repair genes *MLH1* and *MGMT* was shown to be a mechanism underlying the development of microsatellite instability (MSI), a phenotype frequently associated with various human malignancies. Recently, aberrant methylation of *MLH1, MGMT* and MSI were shown to be associated with mutations in genes such as *BRAF, RAS* and *IDH1* in colon and brain tumours. Little is known about the methylation status of *MLH1* and *MGMT* in thyroid tumours and its association with MSI and mutational status.

**Methods:**

In a series of 96 thyroid tumours whose mutational profiles of *BRAF*, *IDH1* and *NRAS* mutations and RET/PTC were previously determined, we investigated *MLH1* and *MGMT* expression and methylation status by qPCR and methylation-specific PCR after bisulphite treatment, respectively. MSI was determined by PCR using seven standard microsatellite markers.

**Results:**

Samples with point mutations (*BRAF*, *IDH1* and *NRAS*) show a decrease in *MLH1* expression when compared to negative samples. Additionally, malignant lesions show a higher MSI pattern than benign lesions. The MSI phenotype was also associated with down-regulation of *MLH1*.

**Conclusions:**

The results of this study allow us to conclude that low expression of *MLH1* is associated with BRAF V600E mutations, RET/PTC rearrangements and transitions (*IDH1* and *NRAS*) in patients with thyroid carcinoma. In addition, a significant relationship between MSI status and histological subtypes was found.

## Background

Thyroid cancer is the most common type of endocrine cancer. Its worldwide incidence has more than doubled since the 1970s. In fact, thyroid http://cancer is the fastest-growing number of new cancer cases in women [1]. Papillary Thyroid Carcinoma (PTC) is the most common subtype, representing approximately 80% of cases. Follicular Thyroid Carcinoma (FTC) is the second most prevalent subtype, accounting for 10-15% of thyroid cancers [1-4].

Multiple genetic and epigenetic alterations have been described in thyroid cancers in recent decades. Most mutations involve effectors of the mitogen-activated protein kinase (MAPK) and phosphatidylinositol 3-kinase (PI3K) pathways. Mutations in *RET/PTC*, *RAS*, or *BRAF*, which result in constitutive MAPK signalling, are found in approximately 70% of PTC cases with little overlap between mutated genes. BRAF V600E is the most common genetic alteration found in PTC, with a worldwide prevalence of 29 to 83% [5-9]. *RET/PTC* rearrangements are the second most common genetic alteration found in PTC. A highly variable rate of *RET/PTC* rearrangement has been reported in different studies; the rate ranges from as low as 0% to as high as 87% [10,11]. Genetic alterations in the PI3K/Akt pathway are more commonly found in the genesis and progression of FTC. *PIK3CA* mutations and amplification were found in FTC. Additionally, PI3K can be activated through genetic or epigenetic inactivation of *PTEN*. Finally, the PI3K pathway can be activated through acquisition of RAS or PAX8/PPAR gamma mutations.

We have previously reported that the BRAF V600E mutation occurs in approximately 48% of PTC cases [7,8]. RET/PTC rearrangements were found in nearly 45% of PTC cases in Brazil (*submitted*). *PIK3CA* and *RAS* mutations were rarely found in our series [12]. Recently, our group [12] and others [13,14] described mutations in the *IDH1* (isocitrate dehydrogenase 1) gene; these mutations were mainly associated with the pathogenesis of the follicular variant of PTC (FVPTC) and FTC but were rarely found in classical PTC.

Microsatellite instability (MSI), caused by defects in the mismatch repair pathway, is a phenotype frequently associated with various human malignancies. Interestingly, promoter hypermethylation of the mismatch repair gene Human *Mut-L* Homologue 1 (*MLH1*) was previously associated with MSI and the presence BRAF V600E mutations in colon cancer [15]. Additionally, hypermethylation of O^6^-methylguanine DNA methyltransferase (*MGMT*), a DNA repair protein that prevents G:C > A:T point mutations by removing alkyl adducts from the O^6^ position of guanine, may lead to *IDH1* and *RAS* mutations in gliomas. Others have described that loss of *MGMT* expression may lead to *PIK3CA* mutations [15]. Whether promoter hypermethylation of the *MLH1* and MGMT genes is the underlying mechanism associated with presence of BRAF V600E, RAS, IDH1, PIK3CA mutations and/or other genetic alterations found in thyroid tumours is still unknown.

In this study, we investigated the methylation status of *MLH1* in a series of benign and malignant thyroid lesions. We next correlated *MLH1* methylation status with expression of *MLH1*, MSI and mutational status. Additionally, as most *IDH1* and *RAS* mutations found in our series of thyroid carcinomas were transitions [12] and considering that an association between *MGMT* and transitions exists, we assessed whether the presence of *IDH1* and *RAS* mutations is associated with *MGMT* methylation and/or loss of *MGMT* expression.

## Methods

### Thyroid samples

A total of 96 thyroid tissue samples obtained from patients who underwent thyroid surgery for thyroid cancer at Hospital São Paulo, Universidade Federal de São Paulo and Hospital das Clínicas, Universidade Estadual de São Paulo was used in this study. All tissue samples were obtained with informed consent according to established human studies protocols at Federal University of São Paulo (protocol 1259/11). To enrich the samples for tumour cells, tissue specimens were obtained from the central part of the tumour specimens. This strategy avoids contamination with surrounding normal tissue and allows for proper pathological diagnosis. Specimens were frozen in liquid nitrogen immediately after surgical resection and stored at −80°C.

Final histological classification was obtained from paraffin-embedded sections. The study included 70 PTCs, 12 FTCs, 7 benign follicular thyroid adenomas (FTAs) and 7 adjacent normal thyroid tissues.

All samples were previously tested for *BRAF, NRAS* and *IDH1* mutations [7,8,12]. *RET/PTC* rearrangements were investigated in 56 PTC samples for which RNA was available (*submitted*).

### Real-time PCR

For *MLH1* and *MGMT* expression analysis, total RNA was isolated using Trizol reagent as described previously (Invitrogen Corporation, Carlsbad, CA, USA) [16]. RNA isolation and cDNA synthesis were performed as previously reported [16,17]. Aliquots of 1 μL of cDNA were used in 12-μL reactions containing SYBR® Green Master Mix (PE Applied Biosystems, Foster City, CA) and 200–250 nM of each primer for the target genes and reference gene (RPS8), as described previously [17]. The primer sequences are described in Table 1.

**Table 1:** Primers used in this study

**Marker or gene**	**Primer (5**^**′**^**-3**^**′**^**)**
**BAT-25**	FW -TCGCCTCCAAGAATGTAAGT
	RV - TCTGCATTTTAACTATGGCTC
**BAT-26**	FW -TGACTACTTTTGACTTCAGCC
	RV -AACCATTCAACATTTTTAACCC
**D5S346**	FW -ACTCACTCTAGTGATAAATCG
	RV-AGCAGATAAGACAGTATTACTAGTT
**BAT40**	FW -GTAGAGCAAGACCACCTT
	RV - AATAACTTCCTACACCACAAC
**D2S123**	FW -AATGGACAAAAACAGGATGC
	RV -CCCTTTCTGACTTGGATACC
**D11S912**	FW -TACTGCTTTGGGTATGCATATG
	RV -GCTTTTTGTCTAGCCATGATTG
**D17S250**	FW -GGAAGAATCAAATAGACAA
	RV -GCTGGCCATATATATATTTAAACC
***MGMT***	FW - CACCACACTGGACAGCCCTTT
	RV - CGAACTTGCCCAGGAGCTTTATTT
***MLH1***	FW -AGAGTGGCTGGACAGAGGAA
	RV -CCCTTCCTCATCAATTTCCA
***RPS8***	FW -AACAAGAAATACCGTGCCC
	RV -GTACGAACCAGCTCGTTATTAG

The reactions were performed in triplicate using a 7500 Real-Time PCR System (PE Applied Biosystems). The threshold cycle (C_t_) for each reaction was obtained using Applied Biosystems Software, and the values were averaged (SD ≤ 1). The PCR efficiencies for *RPS8*, *MLH1*, and *MGMT* were 1.0, 0.99 and 1.0, respectively (data not shown). As PCR efficiencies were comparable, relative expression levels were calculated according to the 2^−∆∆CT^ (ddCt formula) as described previously [8,17].

### DNA extraction and bisulphite treatment

A portion of each tissue was used for the extraction of genomic DNA, which was performed using an adapted phenol-chloroform procedure. One microgram of DNA was treated with sodium bisulphite to convert cytosine to uracil using the EpiTect® Bisulfite kit (QIAGEN, Valencia, CA, USA) according to the manufacturer's recommendations. Briefly, the conversion was made using the following thermal profile: 5 minutes at 95°C, 25 minutes at 60°C, 5 minutes at 95°C, 85 minutes at 60°C, 5 minutes at 95°C, 175 minutes at 60°C and storage at 20°C. The DNA samples were purified, and bisulphite-treated DNA was resuspended in 30 μL of elution buffer for gene methylation analysis.

### DNA methylation analysis

DNA methylation was detected using methylation–specific PCR (MSP) performed with a primer set specific to the methylated or un-methylated sequence (M or U sets, respectively) [18]. The PCR reactions were performed in a final volume of 25 μL, containing approximately 200 ng of sodium bisulphite-treated DNA and 25 pmol of each primer. The PCR amplifications were performed for 30 cycles and consisted of a denaturation step of 95°C for 5 min, a primer-annealing step of 58°C for 35 sec and an extension step at 72°C for 40 sec, with a single final extension step of 72°C for 7 min. The reaction products were separated by electrophoresis on 8% polyacrylamide gels and visualised by silver staining.

### Microsatellite instability analysis

We analysed the microsatellite instability pattern using 7 standard microsatellite markers, of which 3 were mononucleotide repeats (BAT25, BAT26 and BAT40) and 4 were dinucleotide repeats (D2S123, D11S912, D2S346 and D17S250). The MSI analysis was performed by PCR using specific primers (Table 1). PCR was carried out in a total volume of 20 μL, containing 200 ng of DNA, 2.5 μL of 10X PCR Buffer, 1 μM primer, 1.5-2.0 mM MgCl_2_, 200 μM dNTPs and 0.5 U of Taq polymerase (Invitrogen); the products were amplified by 1 cycle of 95°C for 5 min followed by 35 cycles of 95°C for 30 s, 55–58°C for 30 s and 72°C for 15 s, with a final extension at 72°C for 1 min. After the reaction, the samples were denatured using single-strand conformation polymorphism (SSCP) by heating at 95°C for 10 min, and gel electrophoresis was performed on the PCR-amplified products using a 6% polyacrylamide gel containing 6 M urea.

To assess MSI, we compared the band pattern produced after gel electrophoresis of paired PCR reactions containing patient-matched normal and tumour DNA. If the normal and tumour (benign or malignant) PCR amplification products displayed different electrophoretic motilities, the case was scored as positive for MSI. Samples showing instability at one locus were scored as MSI-Low (MSI-L), and those showing instability at two or more loci were scored as MSI-High (MSI-H).

### Statistical analysis

Categorical data were summarised using frequencies and percentages. The relationship between the mutation, methylation and MSI statuses in PTC, FTC or benign subgroups was determined using Fisher’s exact test. Statistical analysis was performed within each subgroup.

For expression analysis, normality was verified using the Shapiro-Wilk normality test. Because the data were not normally distributed, non-parametric statistics were used. A Mann–Whitney test was performed to evaluate the relationship between the expression of *MLH1* or *MGMT* and mutational status (mutant or wild-type) and to evaluate the relationship between MSI or MSS in PTC, FTC or benign subgroups. P values <0.05 were considered significant. The SPSS software (version 11.5; SPSS Inc., Chicago, IL, USA) was used for data analysis.

## Results

### Samples

Of the 82 malignant samples, a BRAF V600E mutation was observed in 29 of 70 (41%) PTC samples. No BRAF V600E mutations were found in the 12 FTC samples (0%). *RET/PTC* was observed in 25 of 56 (45%) PTC samples. *IDH1* mutations were observed in 5 of 70 (7%) PTC samples and 4 of 12 (33%) FTC samples. *NRAS* mutations were observed in 3 of 70 (4%) PTC samples and 3 of 12 (25%) FTC samples. No mutations were found in the benign group.

### Expression of *MLH1* was lower in mutated thyroid carcinoma

In this study, the promoter methylation patterns of two DNA repair genes, *MHL1* and *MGMT*, were evaluated in thyroid samples. In the PTC group, the data showed that *MLH1* and *MGMT* were methylated in 44% and 64% of the cases, respectively. A similar pattern was observed in the FTC subgroup. No significant difference was observed between benign and FTC or PTC samples (Table 2).

**Table 2:** **Association between methylation patterns of *MLH1 *and *MGMT *and histological subtypes**

**Methylation status**		**PTC (n = 70)**		**FTC (n = 12)**		**Benign (n = 14)**
		**N (%)**	p*	**N (%)**	p*	**N (%)**
*MLH1*	Methylated	31/70 (44%)	0.242	4/12 (33%)	0.238	9/14 (64%)
	Unmethylated	39/70 (56%)		8/12 (67%)		5/14 (36%)
*MGMT*	Methylated	45/70 (64%)	0.763	8/12 (67%)	0.701	8/14 (57%)
	Unmethylated	25/70 (36%)		4/12 (33%)		6/14 (43%)

*p values compared against benign group.

We also evaluated the relationship between promoter methylation and presence of specific mutations. For this analysis, we defined negative as those samples that proved to be negative for the panel of mutations, *i.e.*, BRAF V600E, NRAS Q61R, IDH1 mutations and RET/PTC rearrangements. The negative subgroup comprises 19 of 70 (27%) PTC and 6 of 12 (50%) FTC samples. Samples harbouring more than one mutation (n = 10) were also excluded from statistical analysis.

**Figure 1:**
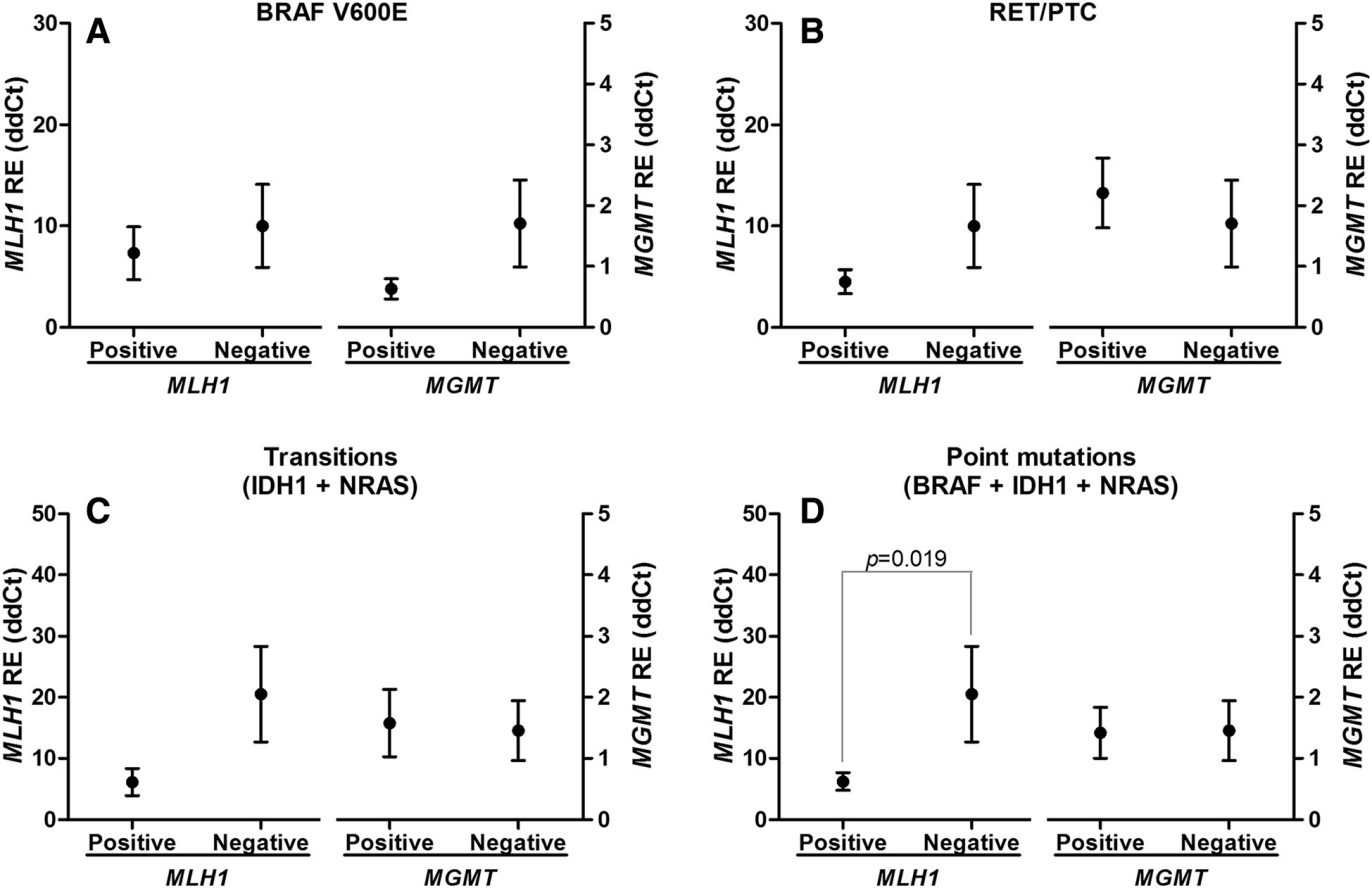
**Relative expression of *MLH1 *and *MGMT *in thyroid cancer samples according to mutational status of BRAF V600E (A), RET/PTC rearrangements (B), samples with IDH1 and NRAS transitions (C) or all samples with point mutations (D).** Analyses of BRAF V600E mutations and RET/PTC were only performed for the PTC group, as these alterations are exclusive of this subtype. The negative group comprises only the samples without any of the alterations investigated. The symbols represent the mean, and the lines represent the standard error. The left Y-axis indicates the relative expression values of *MLH1,* and the right Y-axis indicates the relative expression values of *MGMT*. The *p* values are indicated if statistically significant.

Although not significant, our data showed a decrease in *MLH1* expression in samples harbouring BRAF V600E mutations (Figure 1A), RET/PTC rearrangements (Figure 1B), or transitions (Figure 1C). However, when samples with point mutations were grouped together, *MLH1* expression was significantly decreased (*p* = 0.019; Figure 1D). No significant relationship was found between *MGMT* expression levels and the mutations.

Despite the decreased expression observed, BRAF V600E-mutated samples had a hypomethylated pattern (*p* = 0.048). No significant relationship was found between *MGMT* methylation and the mutations (Table 3).

**Table 3:** **Association between methylation patterns of *MLH1 *and *MGMT *and mutational status**

**Methylation status**		**Mutational status**	**N (%)**	***p***
BRAF V600E*				
*MLH1*	Methylated	Positive	4/20 (20%)	***0.048***
		Negative	10/19 (53%)	
	Unmethylated	Positive	16/20 (80%)	
		Negative	9/19 (47%)	
*MGMT*	Methylated	Positive	14/20 (70%)	0.333
		Negative	10/19 (53%)	
	Unmethylated	Positive	6/20 (30%)	
		Negative	9/19 (47%)	
RET/PTC*				
*MLH1*	Methylated	Positive	8/16 (50%)	1.000
		Negative	10/19 (53%)	
	Unmethylated	Positive	8/16 (50%)	
		Negative	9/19 (47%)	
*MGMT*	Methylated	Positive	9/16 (56%)	1.000
		Negative	10/19 (53%)	
	Unmethylated	Positive	7/16 (44%)	
		Negative	9/19 (47%)	
**Transitions (IDH1 + NRAS)**				
*MLH1*	Methylated	Positive	5/11 (45%)	1.000
		Negative	13/25 (52%)	
	Unmethylated	Positive	6/11 (55%)	
		Negative	12/25 (48%)	
*MGMT*	Methylated	Positive	9/11 (82%)	0.268
		Negative	15/25 (60%)	
	Unmethylated	Positive	2/11 (18%)	
		Negative	10/25 (40%)	
**Point mutations (BRAF + IDH1 + NRAS)**				
*MLH1*	Methylated	Positive	14/41 (34%)	0.199
		Negative	13/25 (52%)	
	Unmethylated	Positive	27/41 (66%)	
		Negative	12/25 (48%)	
*MGMT*	Methylated	Positive	29/41 (71%)	0.426
		Negative	15/25 (60%)	
	Unmethylated	Positive	12/41 (29%)	
		Negative	10/25 (40%)	

* Analysis performed only in the PTC group.

Negative group comprises only samples without any of the alterations investigated.

### Malignant lesions show a higher pattern of MSI

Microsatellite instability analysis showed that 37% of the samples were positive for the D17S250 microsatellite instability marker, 34% for D2S346, 19% for D2S123, 12% for D11S912, 10% for BAT40, 10% for BAT26 and 2% for BAT25.

Our data showed that 84% (59/70) of PTC samples had MSI. Among them, 64% (38/59) showed a MSI-H pattern and 46% (21/59) a MSI-L pattern. In the FTC group, 92% (11/12) of samples had MSI; 82% (9/11) were MSI-H and 18% (2/11) were MSI-L. In the benign group, no MSI-H was observed; all MSI positive samples (43% - 6/14) were MSI-L. Therefore, a significant difference in the MSI patterns between PTC and FTC compared with the benign group was observed (Table 4). Furthermore, no relationship was observed between MSI and any mutations (Table 5).

**Table 4:** Relationships between mutations and MSI patterns by histological subtype

**MSI status**	**PTC**		**FTC**		**Benign**
	**N (%)**	p*	**N (%)**	p*	**N (%)**
**MSI**	59/70 (84%)	***0.002***	11/12 (92%)	***0.012***	6/14 (43%)
MSI-H	38/59 (64%)		9/11 (82%)		-
MSI-L	21/59 (46%)		2/11 (18%)		6/6 (100%)

* *p* values compared against the benign group.

**Table 5:** Relationships between mutations and MSI patterns

** Mutation status**	**N (%)**	***p***
BRAF V600E*		
Positive	18/20 (90%)	1.000
Negative	17/19 (89%)	
RET/PTC*		
Positive	13/16 (81%)	0.642
Negative	17/19 (89%)	
**Transitions (IDH1 + NRAS)**		
Positive	9/11 (82%)	0.631
Negative	22/25 (88%)	
**Point mutations (BRAF + IDH1 + NRAS)**		
Positive	35/41 (85%)	1.000
Negative	22/25 (88%)	

* Analysis performed only in the PTC group.

Negative group comprises only samples without any of the alterations investigated**.**

### Relationship of *MGMT* and *MLH1* expression, methylation and MSI

**Figure 2:**
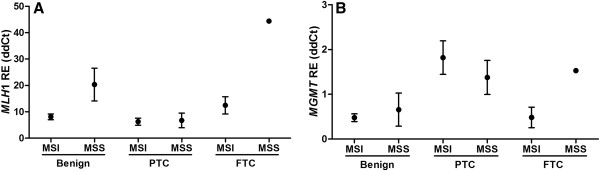
**Relative expression of *MLH1 *(A) and *MGMT *(B) in samples according to MSI and MSS pattern and histological subtype.** The symbols represent the mean, and the lines represent the standard error.

Regarding the effects of *MLH1* and *MGMT* expression on MSI status, our data showed that the MSI phenotype correlated with down-regulation of *MLH1* among patients with benign lesions compared with samples with MSS. Similar results were also observed for *MGMT* (Figure 2A and 2B). As the FTC group only had one sample with MSS, the statistical analysis could not be performed.

Although MSI status was not correlated with *MLH1* expression levels in PTC, we found a marginal association between MSI and *MLH1* methylation in the PTC group (p = 0.079). The methylation pattern of *MGMT* was associated with MSI in PTC samples (p = 0.01; Table 6).

**Table 6:** **Analysis of MSI and *MLH1 *or *MGMT *methylation**

**Methylated and MSI**	**PTC**	**FTC**	**Benign**
	**N (%)**	**N (%)**	**N (%)**
*MLH1*	24/31 (77%)*	3/4 (75%)	4/9 (44%)
*MGMT*	38/45 (84%)**	7/8 (87%)	3/8 (37%)

*p = 0.079 compared with the benign group; **p = 0.01.

## Discussion

DNA repair mechanisms are essential for correcting post-replication errors. Impaired DNA repair is related to increases in mutation frequency, genomic instability and cell death. Aberrant DNA methylation and expression silencing is an important molecular alteration that is commonly detected in DNA repair genes in different types of cancer.

Because *MLH1* promoter methylation was previously associated with MSI and a BRAF V600E mutation [19], we tested the methylation status of *MLH1* in a series of thyroid tumours and correlated them with mutational status and MSI. Additionally, a previous study has reported that *MGMT* hypermethylation was associated with transitions in *IDH1* and *RAS*[20]. Thus, we assessed whether the presence of *IDH1* and *RAS* mutations is associated with *MGMT* methylation and/or loss of *MGMT* expression.

Our study did not find significant differences in *MLH1* and *MGMT* promoter methylation between the groups studied. This result suggests that mechanisms other than DNA methylation in the CpG island studied, e.g., methylation in other CpG islands, miRNAs and histone modifications, could be acting to silence the expression of the genes studied.

The putative relationships between *MLH1* expression and BRAF V600E, RET/PTC and IDH1 genetic alterations were evaluated. Our results showed diminished expression of *MLH1* in patients harbouring BRAF V600E mutations, RET/PTC rearrangements and transitions (*IDH1* and *NRAS*)*.* Although not significant, this result suggests a trend toward significance. Studying a larger set of samples may provide a more detailed understanding of this issue. In fact, when all samples with point mutations were grouped together, a significant association was found. To the best of our knowledge, this evidence is the first to indicate that the down-regulation of *MHL1* is related to BRAF V600E mutations, RET/PTC rearrangements and transitions (*IDH1* and *NRAS*) in patients with thyroid carcinoma.

We did not find a significant correlation between *MGMT* hypermethylation and/or loss of expression and its association with mutational status. A larger sample set may be necessary to reject the hypothesis that *MGMT* hypermethylation or loss of expression is not associated with IDH1 or RAS mutations. Interestingly, the primary mutation found in IDH1 brain tumours was at codon R132, while in thyroid carcinomas, non-R132 mutations were found [12].

Microsatellite instability is a hallmark of the mismatch repair (MMR) deficiency [21,22]. Thus, to evaluate the MSI status of the thyroid samples, we used seven markers. The microsatellite markers demonstrating the highest frequency of MSI were D17S250, D2S346 and D2S123. The lowest frequencies were observed for BAT40, BAT26 and BAT25. Furthermore, mononucleotide markers (BAT40, BAT26 and BAT25) present the lowest frequency of instability among all microsatellite markers tested for both benign and malignant thyroid tumours [23,24].

A significant relationship between MSI status (MSI-H) and histological subtypes was demonstrated in both PTC and FTC, and a higher frequency was found in patients with FTC. MSI is related to malignancy and the clinicopathological factors that indicate poor prognosis [23-25]. MSI does not act as an early event in thyroid tumourigenesis, but MSI is instead involved in tumour progression [24], indicating that MSI might play an important role in thyroid carcinogenesis, as previously described [23-26]. We evaluated the relationship between each mutation and the MSI pattern. Although the BRAF V600E mutation has been associated with sporadic MSI-H colorectal cancers [27], no relationship was found between BRAF V600E mutations, RET/PTC rearrangements and transitions (*IDH1* and *NRAS*) and MSI status.

Although it has been suggested that in thyroid tumours, MSI is an important indicator of defects in the MMR system [28], the data presented in this study showed no relationship between *MLH1* expression and MSI status. In addition, a previous study on colorectal cancer showed that methylation of another DNA repair gene, *MGMT*, is also linked to MSI [22,29]. In thyroid samples, our data showed a relationship between promoter methylation and MSI phenotype.

## Conclusions

Taking these facts into consideration, the results of this study allow us to conclude that low expression of *MLH1* is associated with BRAF V600E mutations and RET/PTC rearrangements and transitions (*IDH1* and *NRAS*) in patients with thyroid carcinoma. Furthermore, a significant relationship between MSI status and histological subtypes was found.

## Abbreviations

PTC: Papillary thyroid carcinoma; FTC: Follicular thyroid carcinoma; FVPTC: Follicular variant of PTC; FTAs: Follicular thyroid adenoma samples; MTC: Medullary thyroid carcinoma; PI3K: Phosphatidylinositol-3 kinase; MAPK: MAP kinase; MMR: DNA mismatch repair; MSI: Microsatellite instability; MSS: Microsatellite stability; MSI-L: MSI-Low; MSI-H: MSI-High; MSP: Methylation–specific PCR.

## Competing interests

The authors declare that they do not have any competing interests.

## Authors’ contributions

JS made significant contributions to the conception and design of the study, acquisition of data, analysis and interpretation of data, drafting and critical revision of the manuscript, statistical analysis, and supervision. She had full access to all of the data in the study and takes responsibility for the integrity of the data and the accuracy of the data analysis. AB made significant contributions to the acquisition of data, critical revision of the manuscript, statistical analysis, and technical support. JC made significant contributions to the acquisition of data, critical revision of the manuscript, and administrative support. MR made significant contributions to the analysis and interpretation of data, critical revision of the manuscript, and statistical analysis. All authors read and approved the final manuscript.

## Pre-publication history

The pre-publication history for this paper can be accessed here:

http://www.biomedcentral.com/1471-2407/13/79/prepub
